# Can Salmonella typhi Cause Acute Appendicitis?

**DOI:** 10.7759/cureus.53213

**Published:** 2024-01-30

**Authors:** Reda H Mithany, Amarah Shaikh, Catherine Scott, Mazin Abdalla, Matthew Graham, Ioannis N Gerogiannis

**Affiliations:** 1 Laparoscopic Colorectal Surgery, Kingston Hospital NHS Foundation Trust, Kingston Upon Thames, GBR; 2 General Surgery, Kingston Hospital NHS Foundation Trust, Kingston Upon Thames, GBR

**Keywords:** appendicectomy, salmonella typhi, terminal ileitis, typhlitis, causes of acute appendicitis, case report, acute appendicitis

## Abstract

Salmonella typhi, commonly known for causing typhoid fever, is recognized as a bacterium responsible for a wide range of gastrointestinal and systemic infections. While its systemic manifestations have been well-documented, its association with localized gastrointestinal complications, such as appendicitis, remains relatively rare and less explored. This case report presents a compelling clinical case of a 55-year-old patient who presented with symptoms of gastrointestinal distress and was diagnosed with S. typhi-induced appendicitis. The patient's history, clinical presentation, laboratory investigations, radiological findings, management, and outcomes are thoroughly discussed. The report also touches upon the broader context of appendicitis etiology and highlights the significance of prompt diagnosis and intervention in cases of Salmonella-induced appendicitis.

## Introduction

Salmonella typhi, a bacterium known for causing typhoid fever, is a well-documented pathogen responsible for a wide range of gastrointestinal and systemic infections. While its systemic manifestations are well-documented, its association with localized gastrointestinal complications such as appendicitis is relatively rare and less explored [1].

Enteric fever remains a global health concern. In 2010, it caused an estimated 26.9 million cases and about 200,000 deaths. Low- and middle-income countries (LMICs) contributed to nearly half of these cases and deaths, with 11.9 million cases and 129,000 deaths. Data from the Global Burden of Disease Study in 2016 estimated around 15.5 million cases and 154,000 deaths from typhoid and paratyphoid fever [2].

Appendicitis ranks among the primary causes of acute abdominal conditions, comprising 7%-10% of emergency presentations. It represents a leading source of lower abdominal pain, prompting numerous visits to the emergency department. Moreover, it remains the predominant diagnosis in hospitalized patients with acute abdominal disorders [3].

This case report aims to present and discuss a rare case of S. typhi-induced acute appendicitis, contributing to the understanding of the association between Salmonella infections and localized gastrointestinal complications, specifically acute appendicitis, and highlighting diagnostic challenges and management considerations.

## Case presentation

Clinical presentation

A 55-year-old previously healthy gentleman presented to the Accident and Emergency Department with a five-day history of progressively worsening abdominal pain, loose stools, and loss of appetite. There was no report of recent travel or consumption of poor-quality food. His past medical, surgical, and family history were unremarkable, and there were no known drug allergies.

On clinical examination, the patient was tachycardic with signs of dehydration such as dry skin and mouth. The vital signs were recorded as follows: heart rate (HR) of 108 bpm, respiratory rate (RR) of 20/min, blood pressure (BP) of 121/80 mmHg, and temperature of 36.2°C. The abdominal examination revealed a soft abdomen and tenderness on deep palpation over the McBurney point, as well as rebound tenderness. Furthermore, Dunphy's, obturator, and psoas signs were positive, with audible bowel sounds.

Laboratory investigations

In the initial laboratory assessments, the patient exhibited abnormal values in various parameters, as summarized in Table 1. The results indicate elevated levels of C-reactive protein, white cell count, and creatinine, along with other deviations from the reference ranges.

**Table 1:** Summary of initial laboratory investigations

Laboratory Parameter	Result	Reference Range
C-reactive protein (CRP)	157 mg/L	<5 mg/L
White cell count (WCC)	15.1 x 10^9/L	4.0-11.0 x 10^9/L
Neutrophils	9.8 x 10^9/L	5-8.0 x 10^9/L
Creatinine	118 μmol/L	60-106 μmol/L
Estimated glomerular filtration rate (eGFR)	59	>90

Microbiology consultation led to the administration of intravenous amoxicillin, gentamicin, and metronidazole for acute appendicitis.

Radiological investigations

**Figure 1:**
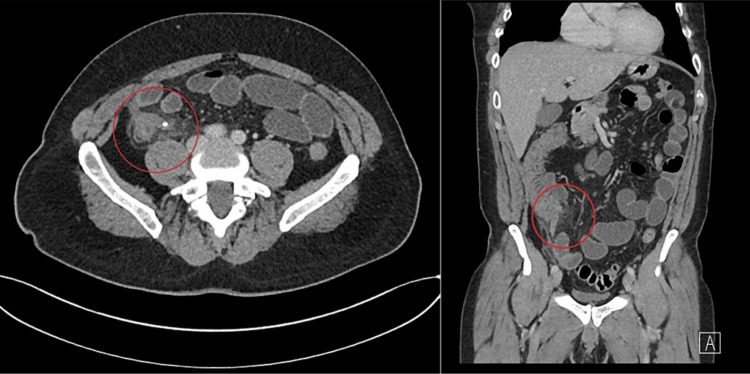
CT of the abdomen and pelvis with contrast confirmed acute appendicitis

In light of the clinical symptoms and laboratory results, a contrast-enhanced CT scan of the abdomen and pelvis was requested. The scan revealed fat strandings around the appendix with an appendicolith in situ. There was no evidence of free fluid or collection in the abdomen, and no other abnormalities were noted (Figure 1).

Management

**Figure 2:**
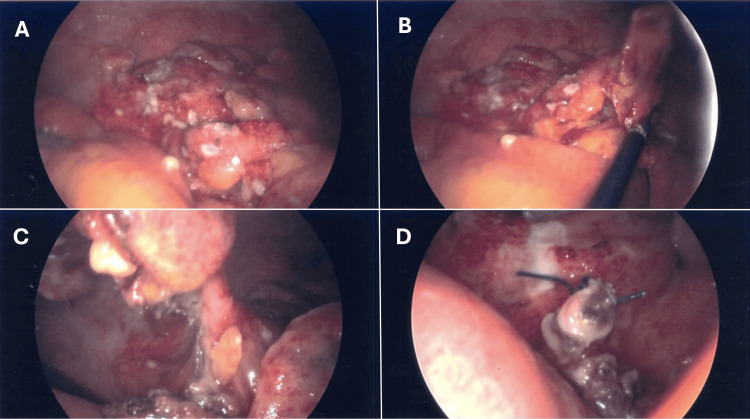
Intraoperatively, the appendix and caecum are both inflamed. A) Inflamed caecum, B) inflamed appendix, C) appendicular base, and D) appendicular stump

A diagnostic laparoscopy and appendicectomy were performed after obtaining written informed consent from the patient. The operation revealed an inflamed and moderately necrotic appendix with a perforation in the middle (Figure 2). The base of the appendix was healthy. Turbid fluid was found in the pelvis, and the right colon was adherent to the lateral abdominal wall. No intra- or immediate post-operative complications were observed.

Cultures of the intra-abdominal fluid showed Salmonella species and Bacteroides fragilis. Therefore, after consultation with the microbiology team, the patient was prescribed a course of oral azithromycin for five days.

During surgery, the patient developed atrial fibrillation (AF), likely triggered by sepsis. Comprehensive investigations were carried out to identify any reversible causes of AF. The patient had experienced palpitations for two months in the past, but had not sought medical attention. With a CHA2DS2-VASc score of zero, anticoagulation treatment was not recommended, and heart rate was controlled with beta-blockers. On the first postoperative day, due to persistent diarrhoea, a stool sample was sent for microbiology culture and sensitivity, which revealed Salmonella species and B. fragilis. The patient clinically and biochemically improved and was subsequently discharged.

Final diagnosis

**Figure 3:**
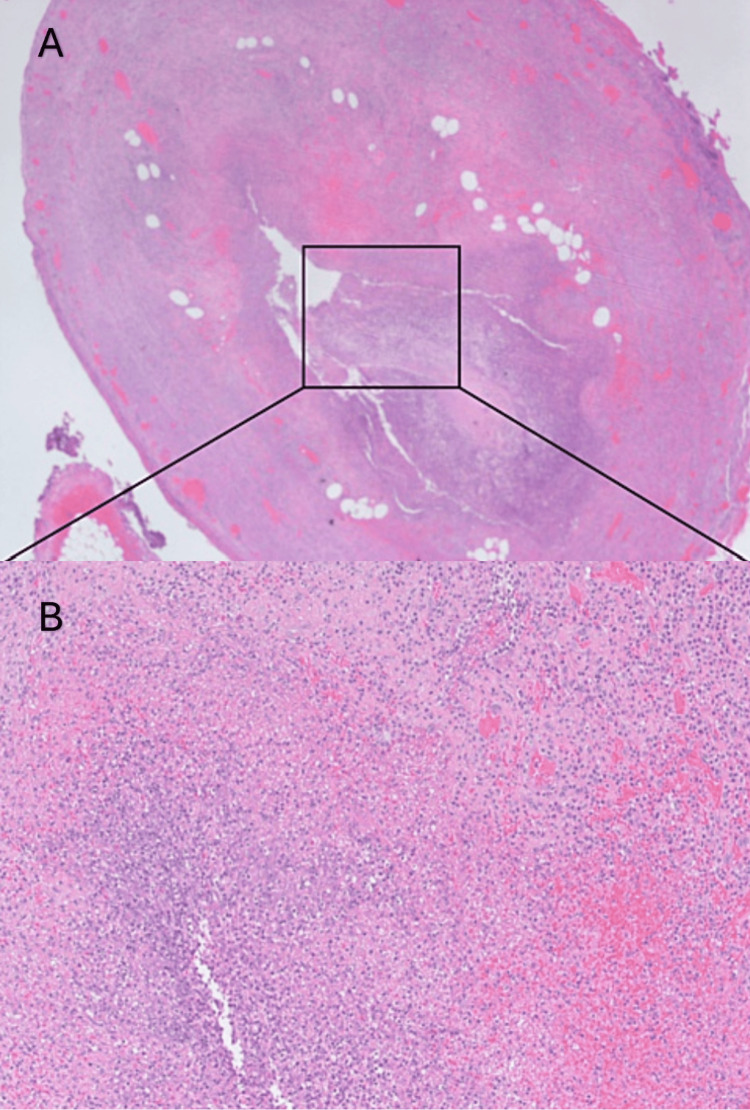
Pathological images of Salmonella typhi infection-related appendicitis A: Lymphoid follicular architecture in the lamina propria shows massive macrophage reactive hyperplasia with small amounts of neutrophil and lymphocytic infiltration. B: A magnification scope of Figure A, which shows macrophage reactive hyperplasia with a small amount of neutrophil and lymphocyte infiltration (hematoxylin-eosin staining, original magnification × 400).

Histopathology findings reported the appendix as measuring 57 x 14 mm, with exudate and attached fat containing firm white material (Figure 3). Microscopic examination revealed transmural acute inflammation indicative of acute appendicitis, with no evidence of dysplasia or malignancy.

## Discussion

The presented case provides an intriguing illustration of Salmonella-induced appendicitis, shedding light on the complex landscape of appendiceal pathology. Appendicitis, traditionally associated with various infectious agents, encompasses a diverse range of microbial culprits, including viruses, bacteria, fungi, and parasites. While the precise aetiology of appendicitis remains enigmatic, mounting evidence suggests potential links between viral illnesses and the development of this condition, prompting inquiries into the relationship between infectious agents and appendicitis [4].

A study by Soltani et al. [5] highlights the intricate association between acute appendicitis and various viruses, including measles virus (MV), influenza virus, dengue fever virus (DFV), human immunodeficiency virus (HIV), human herpesviruses, rotavirus, and adenovirus. These findings emphasize the multifaceted nature of appendicitis aetiology, implicating viral infections as potential contributing factors [5].

In our case, the presence of a fecalith on CT imaging raises questions about its role in appendiceal pathology. However, it is important to recognize that patients can harbor fecaliths without experiencing appendicitis, suggesting the possible involvement of other factors, such as infectious agents. The marked caecal inflammation observed during the operation, combined with the patient's history of prolonged diarrhea and a positive stool culture for Salmonella species, strongly suggests Salmonella as the primary instigator in this case [6].

The association between Salmonella-induced appendicitis and our patient's clinical presentation aligns with previously documented cases described by Sartori et al. [7]. This reinforces the potential role of Salmonella in precipitating acute appendicitis, further supporting our diagnostic assessment [7].

The presence of a fecalith in the appendix emerges as a significant prognostic factor, particularly in cases where nonoperative interventions prove ineffective for complicated appendicitis in adults. This underscores the importance of careful evaluation of fecalith presence in appendicitis diagnosis and management, as it can serve as a critical determinant in treatment decisions [8]. Moreover, the decision to schedule a colonoscopy for this patient within four to six weeks post-appendicectomy is well-founded. The intraoperative finding of an inflamed cecum, coupled with the increased risk of colorectal cancer following appendicitis, emphasizes the importance of vigilant postoperative surveillance. This precautionary measure aligns with findings from a comprehensive retrospective study, reaffirming the imperative for continued surveillance in such cases [9,10].

While fungal infections affecting the appendix are rare, they do occur. Mucormycosis, for instance, has been documented as a cause of inflammatory masses in the lower right abdominal region, affecting the appendix, ileum, and cecum, particularly in patients undergoing chemotherapy [11].

Intriguingly, parasites can also lead to appendicitis. A notable case report describes a 17-year-old Caucasian female who presented with a classic picture of acute appendicitis. Intra-operatively, Enterobius vermicularis, commonly known as pinworm, was found obstructing and traversing the base of the appendix, underscoring the diverse array of etiological factors that can contribute to this condition [12].

There are a few similar case reports to ours, with one showing similarities in CT scan results for acute appendicitis and microbiology findings of S. typhi. However, unlike our case, that report had additional CT findings of spleen and right renal infarctions. Our case stands out because persistent diarrhea led us to thoroughly consider appendicitis etiology, prompting a stool culture to investigate potential enteric pathogens. In addition, diarrhoea can be a misleading symptom, leading to the diagnosis of gastroenteritis rather than acute appendicitis. It emphasizes the importance of considering the patient's overall symptoms, exploring various causes, and considering differential diagnoses [13].

Nonoperative management is appropriate in patients who have a clinical diagnosis of localized appendicitis without physical findings of generalized peritonitis or imaging evidence of large abscess, phlegmon, perforation, or tumor [14]. In our case, the presence of fecalith and the patient’s clinical picture, as well as the inflamed appendix shown on the CT scan, led us to proceed with surgical intervention. The CODA trial results are noteworthy, revealing that, in treating appendicitis, antibiotics demonstrated effectiveness similar to appendectomy using a standard health status measure. Almost 30% in the antibiotic group opted for appendectomy within 90 days. Individuals with an appendicolith faced elevated risks of both appendectomy and complications compared to those without [15].

Summarising, the presence of an uncommon etiology for acute appendicitis does exist and warrants consideration, particularly in cases with persistent and/or atypical symptoms. Diagnostic tools such as CT scans can be useful and can provide additional information to facilitate decision-making in those cases.

## Conclusions

This case report highlights the intricate and less-explored correlation between S. typhi and appendicitis. The presented case imparts significant perspectives on the clinical manifestation, diagnostic complexities, and management of Salmonella-induced appendicitis. The connection between Salmonella and appendicitis is substantiated by comprehensive clinical and laboratory evidence, providing a compelling basis for additional exploration into the potential involvement of infectious agents in the genesis of this condition. As differential diagnosis can be challenging, a low threshold for imaging can be key for successful management.
